# A Disease Register for ME/CFS: Report of a Pilot Study

**DOI:** 10.1186/1756-0500-4-139

**Published:** 2011-05-09

**Authors:** Derek Pheby, Eliana Lacerda, Luis Nacul, Maria de Lourdes Drachler, Peter Campion, Amanda Howe, Fiona Poland, Monica Curran, Valerie Featherstone, Shagufta Fayyaz, Dikaios Sakellariou, José Carlos de Carvalho Leite

**Affiliations:** 1 Buckinghamshire New University, Uxbridge Campus, 106, Oxford Road, Uxbridge, Middlesex, UB8 1NA, UK; 2 London School of Hygiene and Tropical Medicine, Keppel Street, London, WC1E 7HT, UK; 3 University of East Anglia, Norwich, NR4 7TJ, UK; 4 University of Hull, Daisy Building (2nd. Floor) Castle Hill Hospital, Castle Road, Hull, HU16 5JQ, UK; 5 School of Healthcare Studies, Cardiff University, Ty Dewi Sant, Heath Park Campus, Cardiff, CF14 4XN, UK

## Abstract

**Background:**

The ME/CFS Disease Register is one of six subprojects within the National ME/CFS Observatory, a research programme funded by the Big Lottery Fund and sponsored by Action for ME. A pilot study in East Anglia, East Yorkshire, and London aimed to address the problem of identifying representative groups of subjects for research, in order to be able to draw conclusions applicable to the whole ME/CFS population.

While not aiming for comprehensive population coverage, this pilot register sought to recruit participants with ME/CFS in an unbiased way from a large population base. Those recruited are constituting a cohort for long-term follow-up to shed light on prognosis, and a sampling frame for other studies.

**Findings:**

Patients with unidentified chronic fatigue were identified in GP databases using a READ-code based algorithm, and conformity to certain case definitions for ME/CFS determined. 29 practices, covering a population aged 18 to 64 of 143,153, participated.

510 patients with unexplained chronic fatigue were identified. 265 of these conformed to one or more case definitions. 216 were invited to join the register; 160 agreed. 96.9% of participants conformed to the CDC 1994 (Fukuda) definition; the Canadian definition defined more precisely a subset of these. The addition of an epidemiological case definition increased case ascertainment by approximately 4%. A small-scale study in a specialist referral service in East Anglia was also undertaken.

There was little difference in pattern of conformity to case definitions, age or sex among disease register participants compared with subjects in a parallel epidemiological study who declined to participate.

One-year follow-up of 50 subjects showed little change in pain or fatigue scores. There were some changes in conformity to case definitions.

**Conclusions:**

Objective evaluation indicated that the aim of recruiting participants with ME/CFS to a Disease Register had been fulfilled, and confirmed the feasibility of our approach to case identification, data processing, transmission, storage, and analysis. Future developments should include expansion of the ME/CFS Register and its linkage to a tissue sample bank and post mortem tissue archive, to facilitate support for further research studies.

## Background

The myalgic encephalomyelitis/chronic fatigue syndrome (ME/CFS) Disease Register pilot feasibility study, in East Anglia, East Yorkshire, and London, is part of the National ME/CFS Observatory project, funded by the Big Lottery Fund and sponsored by the charity Action for ME. The programme is managed in close liaison with people with ME/CFS and carers.

The study objectives were to:-

• establish a disease register for ME/CFS.

• demonstrate that it can be managed in accordance with legal and ethical requirements.

• assess the effectiveness and comprehensiveness of case ascertainment methods in different communities.

• determine whether or not duplicate entries could be readily detected.

• assess the feasibility of regular follow-up.

• confirm that the data transmission and processing methods were demonstrably secure.

• involve people with ME/CFS and carers in the management of the project.

Disease registers, including the American Veterans' Affairs Gulf War Registry [1,2] and twin registries, have been used to study ME/CFS. Swedish Twin Registry studies showed CFS to be associated with premorbid stress [3-5]. American twin registry studies [6,7] showed that the prevalence of fatiguing illness depended on case definition [8]. A disease-specific twin registry for chronic fatigue has now been established [9].

A 2002 review of disease registers in England [10] asserted that in chronic diseases "... an accurate well-maintained register is a prerequisite to providing comprehensive and coordinated care" [11]. With governmental commitment to establish disease registers in mind [12], the authors identified approximately 250 disease registers recording all cases of a disease in a population, which they distinguished from clinical databases [10]. Our study did not aim at comprehensive population coverage, which would have been unrealistic as many doctors do not recognise the existence of ME/CFS, nor diagnose it [13]. Rather, the study addresses a problem of ME/CFS research, where frequently findings of intervention studies in unrepresentative groups, e.g. excluding severely incapacitated patients, are extrapolated to the whole ME/CFS population. The NICE guidelines on ME/CFS [14] have been criticised on these grounds [15]. Similar problems arise in epidemiological studies [16].

The disease register sought to recruit participants in an unbiased way from a large population. They will be followed up long-term, since little is known about prognosis [17] The register will also constitute a sampling frame for other studies, including intervention studies, to generate results capable of being generalised to the whole ME/CFS population.

## Methods

A descriptive epidemiological study of ME/CFS was carried out in three English regions. General practices in East Yorkshire and East Anglia were invited to participate by the local academic Primary Care Departments in the universities of Hull and East Anglia respectively, and in London by the relevant Primary Care Trust. Patients with unexplained chronic fatigue were identified in GPs' computerised databases by an algorithm identifying READ diagnostic terms indicating probable or possible ME/CFS, while excluding other fatiguing conditions. The primary diagnostic terms (indicating probable ME/CFS) were chronic fatigue syndrome, post viral asthenic syndrome, neurasthenia, fatigue syndrome, post infectious encephalitis, and fibromyalgia. Secondary diagnoses, indicating possible ME/CFS, were 'Tired all the time' (TATT), asthenia, tiredness, fatigue, and neurasthenia or nervous debility. The exclusions were Addison's disease, Cushing's syndrome, hypothyroidism, hyperthyroidism, diabetes mellitus, anaemia, iron deficiency or overload, cancer, rheumatological and auto-immune disorders (rheumatoid arthritis, lupus, polymyositis and polymyalgia rheumatic), AIDS, multiple sclerosis, parkinsonism, myasthenia gravis, B12 deficiency, active infections (tuberculosis, chronic hepatitis), alcohol or substance abuse, sleep apnoea, major psychiatric disorders including bipolar disorder, psychosis and anorexia/bulimia, and major organ failure.

GPs reviewed patients with primary diagnoses to exclude those with symptoms explicable by other diagnoses, or whose participation was contraindicated for personal or clinical reasons. Those with secondary diagnoses were also reviewed. Patients identified were invited to participate in the descriptive epidemiological study, and sent an information sheet and consent form, and symptom assessment instruments. Data was entered locally and transmitted using secure on-line communications to the London School of Hygiene and Tropical Medicine (LSHTM), where a web-based bespoke system was hosted on a UNIX web server using PHP and MySQL database. The system used an encrypted Secure Sockets Layer (SSL) to encrypt data interactions. Personal data was also encrypted. A computerised algorithm was applied to the symptom assessment data to identify subjects who fulfilled at least one of three case definitions, i.e.

(a) The CDC 1994 (Fukuda) definition [18], the most widely used case definition in ME/CFS research,

(b) The Canadian definition [19], recently promulgated and thought to define more precisely patients with unequivocal ME/CFS,

(c) An epidemiological case definition [20], intended to be a robust yet simplified and more inclusive definition of ME/CFS for epidemiological studies. This has two levels, 1, identifying mild to moderate disease, and 2, identifying more severely affected subjects, with a different symptom profile.

Subjects conforming to at least one case definition, unless they did not know or did not accept that they had ME/CFS, were contacted and invited to participate in the Disease Register. A subset of participant data, comprising and GP practice identifiers, contact details, personal characteristics (date of birth, gender, ethnicity), details of consent, and conformity to case definitions, was then held in the LSHTM system.

Participants completed other assessment instruments, including SF-36 [21] and visual analogue scales for pain and fatigue. After one year, a sample of fifty participants was followed up with a further questionnaire, to assess effectiveness of follow-up procedures.

A small-scale study of cases attending a specialist referral service in East Anglia (i.e. covering the area of some participating practices) was also undertaken, to increase the basis of recruitment, and to examine the effectiveness of duplicate entry identification..

For analysis, a severe case is one with (i) tiredness/fatigue most days, (ii) unable to do activities because of tiredness/fatigue, (iii) activities reduced more than 50% since falling ill, (iv) fatigue debilitating and affecting mental and physical functioning, and (v) pain score eight out of ten, and/or fatigue score eighty out of a hundred, or more.

The study was approved by the London Multi-Centre Research Ethics Committee, and the Ethics Committee of LSHTM.

## Results

The study reviewed case ascertainment methods, procedures for handling duplicate registrations, validity and appropriateness of primary care-based data collection methods in ethnically and socially diverse populations, follow-up arrangements, and effectiveness and legal and ethical compliance of data management, including data access, data security, and monitoring data quality, i.e. completeness, comprehensiveness, accuracy and timeliness. We established arrangements for accountability, reporting and publicity and a clinical network to support the work of the register. These are considered in the 'Discussion' section.

The 29 participating practices covered a population aged 18 to 64 inclusive of 143,153. Five practices were in East Anglia and five in London. There were nineteen, on average smaller, practices in East Yorkshire. Among this population, 510 patients with unexplained chronic fatigue were identified, and 265 conformed to one or more case definitions. 216 were invited to participate in the register, and 160 agreed.

### Conformity to Case Definitions

**Figure 1:**
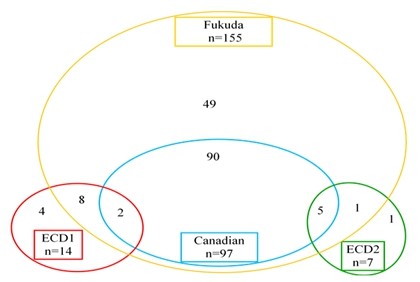
**The inter-relationship of diagnostic definitions used in the ME/CFS Disease Register Pilot Study**.

Figure 1 illustrates distribution by case definition. Most cases (96.9%) fulfilled the CDC 1994 (Fukuda) definition, while the Canadian definition defined more precisely a subset of these. Use of the epidemiological case definition increased overall ascertainment by nearly 4%.

There was little difference in conformity to case definitions between disease register participants, non-participants or epidemiological study subjects overall (Table 1).

**Table 1:** Conformity of Epidemiological Study and Disease Register Participants to Case Definitions

Case Definition	Epidemiological Study				Disease Register	
			
	All subjects		Subjects not in disease register			
	**No. cases**	**% total (95%** **confidence interval)**	**No. cases**	**% total (95%** **confidence interval)**	**No. cases**	**% total (95%** **confidence interval)**

Fukuda	282	96.9 (94.8 - 99.0)	127	96.9 (93.9 - 100.0)	155	96.9 (94.1 - 99.7

Canadian	154	52.9 (44.9 - 61.0)	57	43.5 (30.4 - 56.6)	97	60.6 (50.7 - 70.5)

TOTAL	291	100.0	131	100.0	160	100.0

Of five cases conforming to the epidemiological case definition but not to the CDC (1994) definition, three manifested only three of the 1994 CDC definition's minor criteria, not the required four, one had other illnesses which were exclusions from the CDC 1994 definition, while the fifth had illness of indefinite onset.

Cases meeting the Canadian definition were compared with CDC (1994) positive cases that did not (Table 2). There were no significant differences in age or sex distribution. None of the participants who reported fatigue less frequently than every day, or who did not regard their fatigue as debilitating, conformed to the Canadian definition. Those conforming to the Canadian definition tended to report a greater impact of fatigue on activities, a greater reduction in activity levels since falling ill, higher pain levels, and higher fatigue levels on recruitment.

**Table 2:** Fukuda definition-positive subjects conforming or not conforming to the Canadian case definition

	Cases fulfilling case definition (n = 122)		Cases not fulfilling case definition (n = 87)		95% confidence interval (difference in percentages)
	**No.**	**% total**	**No.**	**% total**	

Age Group: < 25	1	0.8	4	4.6	-8.6 - 1.0
25-34	15	12.3	6	6.9	-2.7 - 1.5
35-44	32	26.2	19	21.8	-7.5 - 1.3
45-54	43	35.2	28	32.2	-10.2 - 1.3
55-64	27	22.1	30	34.5	-25.0 - 0.3
65+	4	3.3	1	1.1	-1.8 - 6.1

Sex: Male	25	20.5	15	17.2	-7.7 - 14.2
Female	97	79.5	72	82.8	-14.2 - 7.7

How often do you have tiredness/fatigue?					
Occasionally	0	0.0	1	1.1	-3.4 - 1.1
< 3 times/week	0	0.0	7	8.0	**-13.9 - -2.2**
Every day or nearly every day	122	100.0	79	90.8	**3.0 - 15.4**

Were your activities (personal, social, professional, at home) affected by the symptoms of tiredness/fatigue?					
Not at all	0	0.0	0	0.0	0.0- 0.0
A little	0	0.0	0	0.0	0.0- 0.0
Needed to reduce	11	9.0	36	41.4	**-44.1 - -20.6**
Can no longer do	111	91.0	51	58.6	**20.6 - 44.1**

Would you say that your activities were reduced to < 50% than before you fell ill?					
Yes, by > 50%	116	95.1	70	80.5	**5.3 - 24.0**
No, by 50% or less	4	3.3	10	11.5	**-15.8 - -0.7**
Don't know	2	1.6	6	6.9	-11.2 - 0.6

Would you say that your fatigue is debilitating and affects your mental and physical functioning?					
Yes	122	100.0	79	90.8	**3.0 - 15.4**
No	0	0.0	4	4.6	**-9.1 - -0.1**
Don't know	0	0.0	3	3.4	-7.4 - 0.5

Pain level (where 0 = no pain, and 10 = pain as bad as possible)					
Mean	6.0		4.6		
95% confidence interval	5.6 - 6.4		2.5 - 3.1		
Standard deviation	2.4		2.8		
Median	6		5		
Interquartile range	5-8		2-7		
No. reporting no pain	2		9		
No. reporting maximum pain	5		3		

Fatigue level on recruitment (where 0 = no problem, and 100 = the most severe health impediment)					
Mean	65.7		54.3		
95% confidence interval	61.0 - 70.4		50.0 - 58.6		
Standard deviation	26.1		21.0		
Median	70		50		
Interquartile range	50-80		50-70		
No. reporting no fatigue	2		2		
No. reporting max. possible fatigue	15		1		

Table 3 compares age group and sex distribution, and table 4 levels of severity, in Register participants and non-participants.

**Table 3:** Age and Sex Distribution among Disease Register Invitees, Participants, and Those Declining Invitation

	All Invitees		Not accepting Invitation		Disease Register Participants	
	
	No. cases	% total (95% confidence interval)	No. cases	% total (95% confidence interval)	No. cases	% total (95% confidence interval)
*Sex*						

Male	42	19.4 (7.2 - 31.7)	8	14.3 (0.0 - 39.0)	34	21.3 (7.2 - 35.3)

Female	174	80.6 (74.6 - 86.6)	48	85.7 (75.6 - 95.8)	126	78.8 (71.5 - 86.0)

*Age group*						

< 25	26	12.3 (0.0 - 24.8)	6	10.7 (0.0 - 36.0)	20	12.5 (0.0 - 27.3)

25-44	73	33.8 (22.7 - 44.9)	23	41.1 (20.6 - 61.6)	50	31.3 (18.1 - 44.4)

45-64	132	61.1 (52.6 - 69.6)	32	57.1 (39.6 - 74.6)	100	62.5 (52.8 - 72.2)

65+	6	2.8 (0.0 - 16.2)	1	1.8 (0.0 - 29.3)	5	3.1 (0.0 - 18.7)

*Ethnicity*						

White British	157	72.6 (65.6 - 79.8)	12	21.4 (0.0 - 45.1)	145	90.6 (85.8 - 95.5)

Other	12	5.6 (0.0 - 18.8)	1	1.8 (0.0 - 28.3)	11	6.9 (0.0 - 22.1)

Not stated	47	21.8 (9.7 - 33.8)	43	76.8 (63.9 - 89.7)	4	2.5 (0.0 - 18.1)

*Region*						

London	29	13.4 (0.8 - 26.1)	16	28.6 (6.0 - 51.2)	13	8.1 (0.0 - 23.3)

East Anglia	81	37.5 (26.7 - 48.3)	15	26.8 (3.9 - 49.7)	66	41.3 (29.1 - 53.4)

Yorks./Humberside	105	48.6 (38.9 - 58.4)	25	44.6 (24.8 - 64.5)	80	50.0 (38.8 - 61.2)

Not stated	1	0.4 (0.0 - 14.9)	-	0.0	1	0.6 (0.0 - 16.4)

TOTAL	216	100.0	56	100.0	160	100.0

**Table 4:** Severity distribution among disease register participants and non-participants

	Participants (n = 158)		Non-Participants (n = 58)		95% confidence interval (difference in percentages)
	**No.**	**% total**	**No.**	**% total**	

How often do you have tiredness/fatigue?					
Occasionally	1	0.6	0	0.0	-0.6 - 1.9
< 3 times/week	6	3.8	2	3.4	-5.3 - 6.0
Every day or nearly every day	151	95.6	56	96.6	-6.8 - 4.8

Were your activities (personal, social, professional, at home) affected by the symptoms of tiredness/fatigue?					
Not at all	0	0.0	0	0.0	0.0 - 0.0
A little	1	0.6	0	0.0	-0.6 - 1.9
Needed to reduce	35	22.2	13	22.4	-13.1 - 12.5
Can no longer do	122	77.2	45	77.6	-13.2 - 12.5

Would you say that your activities were reduced to < 50% than before you fell ill?					
Yes, by > 50%	144	91.1	48	82.8	-2.5 - 19.3
No, by 50% or less or not at all	11	7.0	5	8.6	-10.1 - 6.8
Don't know	3	1.9	5	8.6	-14.4 - 1.0

Would you say that your fatigue is debilitating and affects your mental and physical functioning?					
Yes	153	96.9	55	94.8	-4.4 - 8.5
No	3	1.9	1	1.7	-3.9 - 4.2
Don't know	2	1.3	1	1.7	-4.3 - 3.4

Pain level (where 0 = no pain, and 10 = pain as bad as possible)					
Mean	5.3		5.6		
95% confidence interval Standard deviation	4.9 - 5.7		4.5 - 6.7		
Median	2.5		2.1		
	6		6		

Fatigue level on recruitment (where 0 = no problem, and 100 = the most severe health impediment)					
Mean	66.0		66.8		
95% confidence interval	63.0 - 69.0		55.6 - 78.0		
Standard deviation	18.3		38.0		
Median	60 < 70		60 < 70		

80.0% of males approached agreed to participate, and 72.4% of females. Subjects from East Anglia were most likely to agree, and those from London least likely. Those not participating tended to report more severe symptoms than register participants, but this was not statistically significant.

Duration of illness prior to recruitment varied from 18 months to 27 years, with a mean of 127.3 months (standard deviation = 84.1 months), and a median of 108 months. There was no difference between males (mean duration prior to recruitment = 128.1 months) and females (mean duration prior to recruitment = 127.1 months) in this respect. The results regarding duration of illness prior to recruitment are summarised in table 5.

**Table 5:** Duration of illness prior to recruitment

Duration (years)	Males		Females		All Participants	
	**No. cases**	**% total**	**No. cases**	**% total**	**No. cases**	**% total**

< 2	-	0.0	1	1.3	1	1.0

2 < 5	2	8.7	18	22.8	20	19.6

5 < 10	10	43.5	23	29.1	33	32.3

10 < 20	9	39.1	25	31.6	34	33.3

20+	2	8.7	12	15.2	14	13.7

TOTAL	23	100.0	79	100.0	102	100.0

### Follow-Up Results

Pain and fatigue levels were recorded in fifty disease register subjects followed up after one year. Pain was assessed on a scale of 0 to 10, where 0 indicated no pain and 10 maximum pain. Fatigue scores ranged from 0 to 100, where 0 indicated no fatigue with exercise and 100 indicated maximum fatigue, bedridden, and unable to self-care. There was little change in either. The mean pain score on recruitment was 4.9, (median 5.0, interquartile range 2.0-7.0; n = 50), and after one year the mean was 5.0 (median 5.7, interquartile range 2.6-7.0; n = 49, p (paired t-test) = 0.66). The mean fatigue score on recruitment was 61.6 (median 60, interquartile range 50-70; n = 49), and after one year was 59.6 (median and interquartile range unchanged; n = 46).

Conformity to case definitions was assessed on follow up. Numbers of subjects conforming to CDC (1994) and Canadian definitions were reduced compared with recruitment, though conformity to case definitions was unchanged for 38 respondents (76%). At recruitment, 49 subjects conformed to the CDC (1994) definition, but only 44 on follow up. 31 subjects conformed to the Canadian definition on recruitment; four no longer conformed at follow-up, but three additional subjects did.

## Discussion

### Objective-Based Evaluation

The case ascertainment methods worked effectively, in different communities, while the secondary care study showed duplicate entries could be readily detected. Regular follow-up is feasible, although a larger scale study is needed to assess drop-out rates. A disease register for ME/CFS can be established and managed in compliance with legal and ethical requirements. Our data transmission and processing methods are demonstrably secure. Researchers have used the register to identify participants for other studies, e.g. of gene expression. Little data is missing, but case ascertainment is not comprehensive; this was not achievable in the particular circumstances of ME/CFS. We established effective project management, including participation by people with ME/CFS and carers in the Project Steering Group.

### Interpretation of Statistical Findings

The results confirm that the Canadian definition [19] defines a subset of cases conforming to CDC(1994) [18]. Use of both definitions enabled us to take advantage of the sensitivity of the former and specificity of the latter.

We attempted to validate the epidemiological case definition [20]. Use of this as an adjunct to the CDC 1994 definition does mitigate under-ascertainment, but it is less inclusive than hoped. It includes some cases excluded by the CDC 1994 definition, but excludes many cases who do meet its requirements.

Disease register participants appear similar to descriptive epidemiological study cases in proportions conforming to various case definitions. Register participants are rather older on average than descriptive epidemiological study subjects, with a rather higher proportion of males. More than three-quarters of disease register participants were female. The modal age (nearly two-thirds of respondents), was 45-64, whereas previous research has suggested a modal age of 25-44 [22]. This may indicate a cohort effect.

Conformity to case definitions varied through time, suggesting that period prevalence rather than point prevalence may be appropriate in descriptive epidemiological studies. Use of formal definitions to identify cases of a syndrome is unsatisfactory, because boundaries are arbitrary and overlap with other syndromes [23], and different case definitions produce different findings in ME/CFS [24,25]. Until phenotypes are defined in terms of underlying pathology, this is unavoidable. However, this does not impede the register's purpose, to undertake long-term follow-up, and create a sampling frame for further studies.

### What this study adds

This is the first systematic attempt to develop a population-based disease register specific to ME/CFS. Participation was voluntary, and we depended on GPs for recruitment of subjects. Many GPs remain reluctant to diagnose ME/CFS [13]. Furthermore, reliance on normative case definitions to determine eligibility for inclusion may result in under-ascertainment, as conformity varies over time.

The study confirmed the feasibility of our methods of case identification, data processing, transmission, storage and analysis, and demonstrated the potential of GP electronic records for identifying patients suitable for registration. Our study met Newton and Garner's requirements [10] of robust and appropriate case definitions, unbiased case ascertainment, and procedures for identifying duplicates and for follow-up.

### Future Developments

For the future, we propose to continue to recruit to the register and to develop linked infrastructure facilities, including a tissue sample bank, to which register participants will be invited to contribute blood samples, e.g. to facilitate nested case-control studies of particular outcomes, with access to stored biological material and detailed follow-up data. A *post mortem *tissue archive is also proposed, and disease register participants will be invited to make advance declarations of willingness to contribute tissues after death.

Other complementary initiatives include the National Outcomes Database, an important infrastructure facility which collates patient data from NHS ME/CFS Collaborative clinical services[26]. This, though larger, differs significantly from the disease register. It is based on secondary care referrals, lacks a population base, and uses the broader case definition advocated by NICE [27].

Extending the use of the disease register as a sampling frame will require capacity to flag records indicating involvement in particular studies, to define additional data fields, and to link records to records in other databases. For outcomes assessment, a disease-specific patient-reported outcome measure (PROM) is needed, but meanwhile the London Handicap Scale [28], a six-item validated instrument which facilitates inter-group comparisons, may be useful [29].

Extending the register to national coverage will require a major system upgrade, possibly involving a multiple tier architecture, including an application server to facilitate remote access for data collection and interrogation, a backend database server, and an offline data store to warehouse captured data. It also requires a web services API (Application Programming Interface) using XML, enabling authorised users to perform validated data submission as well as certain analyses of aggregated data from remote locations, to minimise data input errors and increase usability.

## Conclusions

ME/CFS is a complex condition. This Disease Register pilot study has validated the methods used to set it up and has provided the basis for a range of initiatives to develop the evidence base needed to understand causes, clinical interventions and access to social support needed to address this challenging disease.

## List of Abbreviations

ME/CFS: Myalgic encephalomyelitis/chronic fatigue syndrome; GP: General practitioner; CDC: Centers for Disease Control; SF-36: Short Form 36; LSHTM: London School of Hygiene and Tropical Medicine; NICE: National Institute for Health and Clinical Excellence; PROM: Patient Reported Outcome Measure; API: Application Programming Interface; XML: Extensible Markup Language;

## Competing interests

The authors declare that they have no competing interests.

## Authors' contributions

DP was overall Project Coordinator for the National ME/CFS Observatory project, and Project Lead for the Disease Register sub-project. EL was Research Manager for the Disease Register, with responsibility for overall management of the sub-project. EL and LN were responsible for the development of the database and the associated algorithms. EL, LN, MD, PC, AH, FP, JL, MC, and VF were involved in design of the project, liaison with general practices, data collection, preparation, storage and transmission. SF and DS were research administrators at LSHTM, and responsible for day-to-day administration of the project. All authors were involved in the preparation of this report, and read and approved the final manuscript.
